# Case Report: Successful treatment of severe fever with thrombocytopenia syndrome associated with hemophagocytic lymphohistiocytosis by preemptively using favipiravir and methylprednisolone

**DOI:** 10.3389/fmed.2025.1566719

**Published:** 2025-06-13

**Authors:** Liang Qiao, Ting-juan Zhang, Yuan Feng, Lei Yang, Ping Cai, Su-wan Liu, Yong-hui Ji, Jun Qian, Jing-dong Zhou

**Affiliations:** ^1^Department of Hematology, The Affiliated People’s Hospital of Jiangsu University, Zhenjiang, Jiangsu, China; ^2^Institute of Hematology, Jiangsu University, Zhenjiang, Jiangsu, China; ^3^Zhenjiang Clinical Research Center of Hematology, Zhenjiang, Jiangsu, China; ^4^The Key Lab of Precision Diagnosis and Treatment in Hematologic Malignancies of Zhenjiang City, Zhenjiang, Jiangsu, China; ^5^Laboratory Center, The Affiliated People’s Hospital of Jiangsu University, Zhenjiang, Jiangsu, China

**Keywords:** severe fever with thrombocytopenia syndrome, hemophagocytic lymphohistiocytosis, preemptive treatment, favipiravir, case report

## Abstract

**Background:**

Severe fever with thrombocytopenia syndrome (SFTS) is an emerging infectious disease caused by a novel Bunyavirus with a high mortality rate. Cytokine storm could be regarded as an important feature of SFTS patients. Severe or critical cases of SFTS are often complicated with the presence of hemophagocytic lymphohistiocytosis (HLH), resulted in rapid disease progression and extremely adverse prognosis. Currently, effective treatments for these severe cases remain unavailable. Herein, we reported a case of SFTS associated with HLH successfully treated by favipiravir and methylprednisolone.

**Case presentation:**

A 73-year-old male farmer was admitted to our hospital with a fever, fatigue, and bicytopenia for 1 week on June 20, 2024. The patient had a history of farm work. The diagnosis of SFTS was confirmed by detection of Bunyavirus RNA in the blood samples. Bone marrow examination revealed marked infiltration of macrophages with hemophagocytosis in the bone marrow leading to a diagnosis of HLH. Notably, the patient was preemptively treated with favipiravir together with methylprednisolone and supportive therapy before the diagnosis of SFTS associated with HLH. Excitingly, the patient was recovered after 10 days treatment.

**Conclusion:**

Early recognition of SFTS complicated with HLH is particularly important, and the preemptive application of favipiravir may improve the prognosis of these patients.

## Background

Severe Fever with Thrombocytopenia Syndrome (SFTS) is a newly emerging infectious disease caused by a novel bunyavirus mainly transmitted by ticks (1). Patients with SFTS can exhibit a variety of symptoms, including fever, muscle pain, and fatigue, while critically ill patients may even exhibit bleeding tendencies, multiorgan failure, and impaired consciousness leading to death (1). The mortality rate in SFTS patients ranges from 2.7 to 45.7%, with an average of 20% (2, 3). Cytokine storm could be regarded as an important feature of SFTS patients (1). Severe or critical cases of SFTS are often complicated with the presence of hemophagocytic lymphohistiocytosis (HLH), resulted in rapid disease progression and extremely adverse prognosis (1, 4). Currently, effective treatments for these severe cases remain unavailable. Favipiravir is an antiviral drug targeting many RNA viruses by inhibiting viral RNA-dependent RNA polymerase selectively or inducing RNA transversion mutations to produce a nonviable viral phenotype (5). Notably, in Japan, Favipiravir has been approved by Pharmaceuticals and Medical Devices Agency (PMDA) in 2024 to treatment SFTS. Herein, we reported a case of SFTS associated with HLH who succeeded in recovery from preemptive treatment by favipiravir and methylprednisolone.

## Case presentation

A 73-year-old male patient was admitted to the emergency department of our hospital with fatigue for one week, fall down with urinary incontinence for one day on June 20, 2024. The patient showed muscle soreness, poor mental status, less talking, but no headache, chest tightness or pain, dizziness, and vomiting. Notably, the patient had a history of daily farm work, but reported no history of tick bite. However, the patient did not measure his temperature after feeling unwell. Routine blood test showed the presence of leukocytopenia and thrombocytopenia with white blood cell (WBC) count 1.57 × 10^9^/L and platelet (PLT) count 62 × 10^9^/L. Biochemical test determined the elevation of blood urea nitrogen (BUN) 13.29 mmol/L, creatinine (CREA) 151 μmol/L, aspartate aminotransferase (AST) 130 IU/L, lactic dehydrogenase (LDH) 564 IU/L, creatine kinase (CK) 407 IU/L. Because of the presence of bicytopenia, the patient was sent to the department of hematology for hospitalization. On admission, his peak body temperature was 38.5°C and normal for pulse, respiratory, and blood pressure. Laboratory tests of routine urinary showed proteinuria (2+), microhematuria (1+) and cylindruria (1+). The serum lipid showed normal for triglyceride (2.26 mmol/L), and serum liver enzymes for alanine aminotransferase (ALT) was increased (63 u/L). A coagulation test indicated normal fibrinogen of 2.53 g/L. Importantly, the serum ferritin level was increased to 4,565 ng/mL. Moreover, the cytokines and chemokines were also elevated in interleukin (IL)-6 15.84 pg./mL, IL-10 9.78 pg./mL, tumor necrosis factor (TNF)-*α* 6.74 pg./mL, interferon (IFN)-*γ* 51.4 pg./mL, IFN-α 288.84 pg./mL. Taken together, the patient was highly suspected as SFTS, and the detection of Bunyavirus RNA in the blood samples was performed.

**Figure 1:**
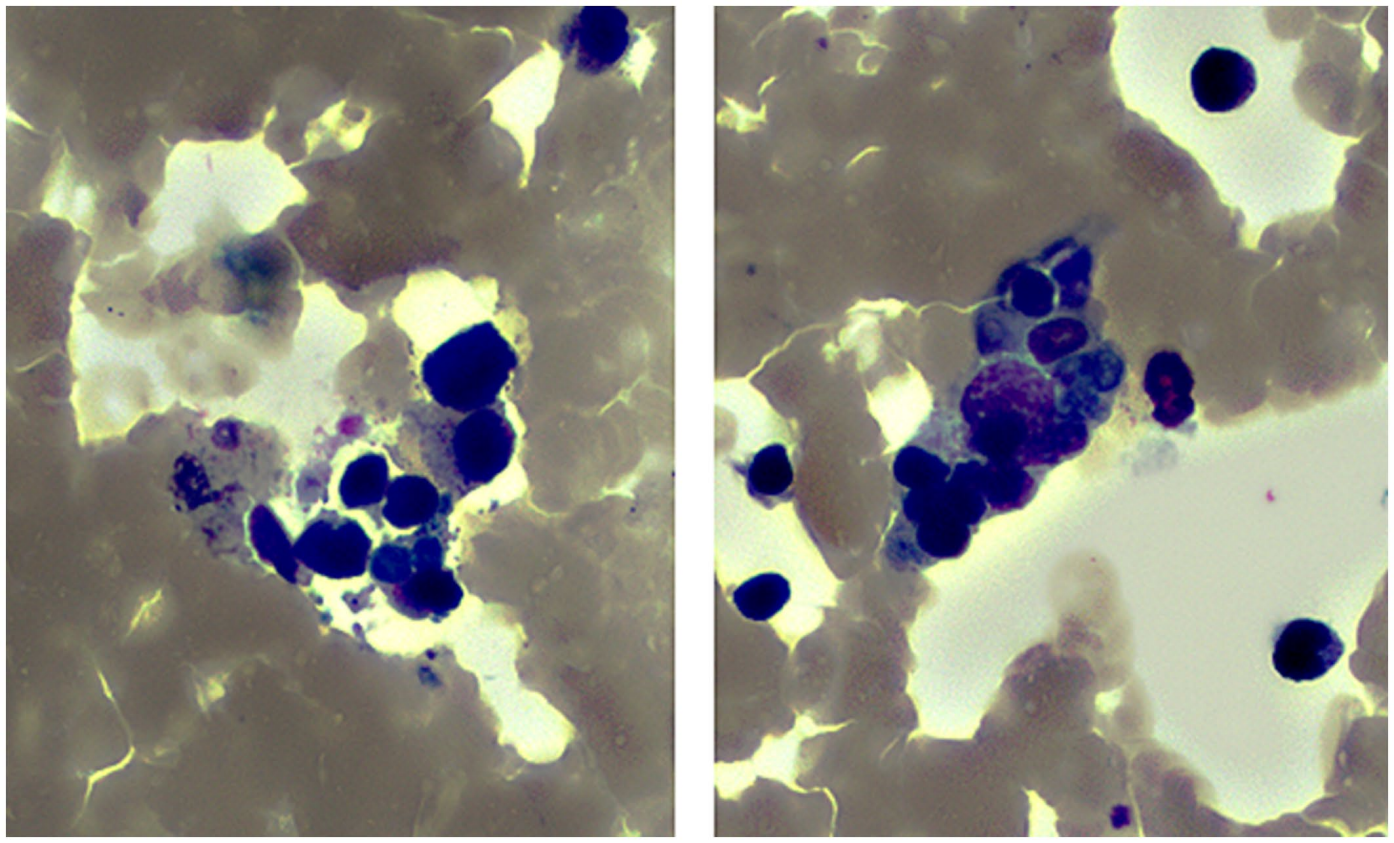
Presence of hemophagocytosis in bone marrow of the patient (×1,000).

During hospitalization, the patient’s fibrinogen was decreased to 1.82 g/L and triglyceride increased to 4.56 mmol/L together with a reduction of WBC and PLT counts. Consequently, bone marrow examination was performed, and revealed marked infiltration of macrophages with hemophagocytosis in the bone marrow (Figure 1), leading to a diagnosis of HLH. On Day 7 of admission, the detection of Bunyavirus RNA by real-time quantitative polymerase chain reaction showed positive with a copy number of 2.69 × 10^3^ TCID50/mL. Finally, the patient was diagnosed with SFTS complicated with HLH.

**Figure 2:**
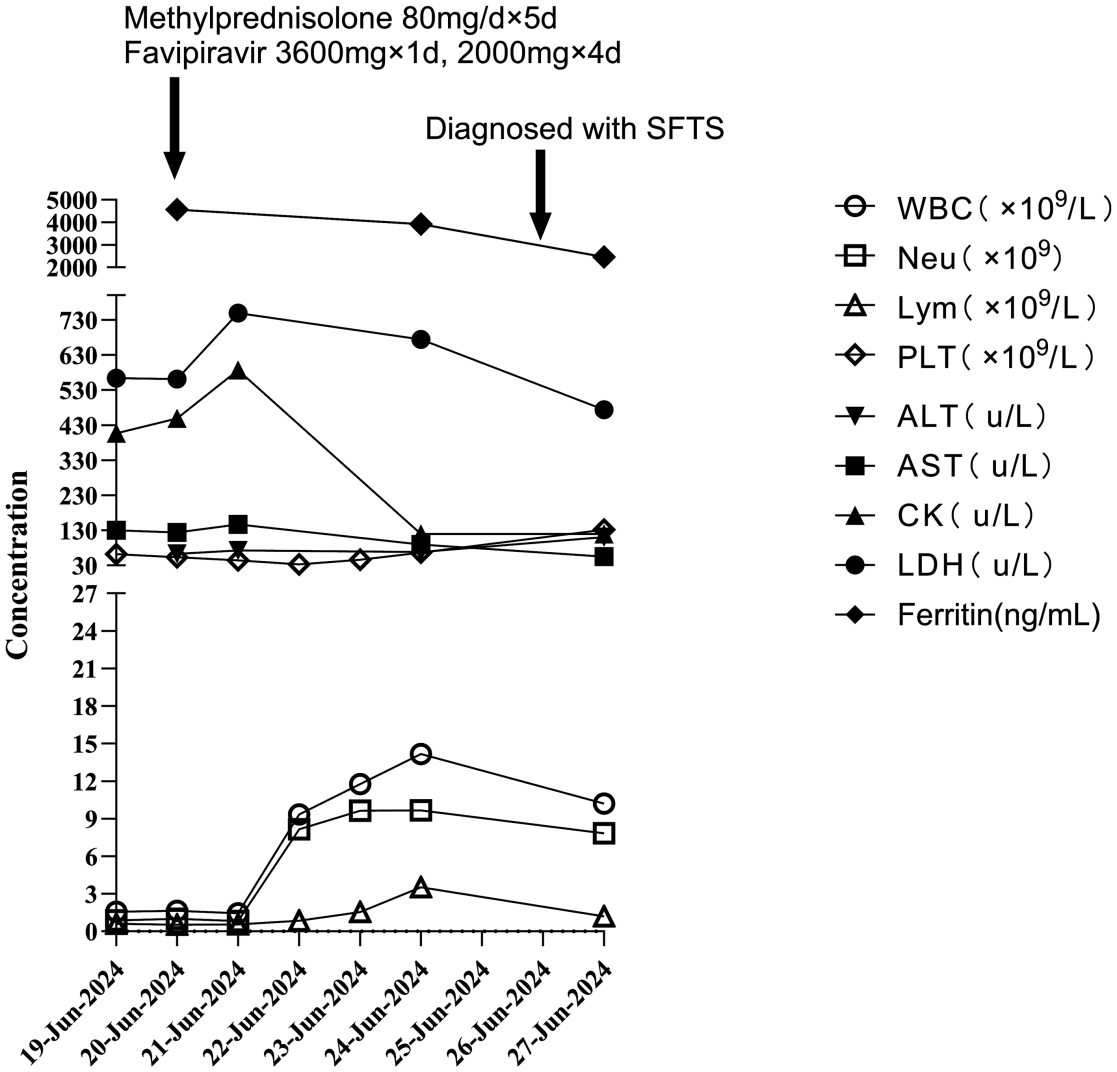
Changes of laboratory parameters in the clinical course of the patient.

Notably, the patient was preemptively treated with favipiravir (3,600 mg d1, 2000 mg d2-d5) together with methylprednisolone (80 mg/d d1–d5, 40 mg/d d6–d7, 20 mg/d d8–d9) and supportive therapy on admission before the diagnosis of SFTS associated with HLH. After active treatment, the patient’s mental state gradually recovered, body temperature was normal, and there were no involuntary muscle tremors throughout the body. The overall improvement of various laboratory indicators was observed in the follow-up examination (Figure 2), and the patient was discharged on June 30, 2024.

## Discussion and conclusion

Although elevated cytokines and chemokines is an important pathophysiological feature in patients with SFTS, severe cases presented with cytokine storm complicated with HLH (1). The prognosis of these patients remains extremely poor, which may be attributed to the cytokine storm and immune dysfunction (4, 6). Previously, a serious of case reports also indicated the rapid death endpoint of SFTS patients with HLH (4, 7). Herein, we reported a case of SFTS associated with HLH who succeeded in recovery from preemptive treatment by favipiravir and methylprednisolone. As is well known, there are no effective vaccines or specific treatments for SFTS, early diagnosis and accurate severity assessment are crucial. However, the detection of Bunyavirus RNA in China usually needs five workdays or more, which markedly increased the difficulty of clinical treatment. Different from previous reports, we preemptively gave the treatment with favipiravir and methylprednisolone for the patient when was highly suspected as SFTS with HLH.

Favipiravir is a purine nucleic acid analogue that is converted into its active phosphoribosylated form within cells and recognized as a substrate by viral RNA dependent RNA polymerase, thereby inhibiting viral replication and transcription (5). Previous studies have revealed the antiviral effect of favipiravir in treating Bunyavirus infection in a mouse lethal model (8). Subsequently, the efficacy of favipiravir in the treatment of SFTS patients has been reported (9, 10). Although a recent systematic meta-analysis demonstrates that antiviral drugs (ribavirin and favipiravir) used routinely in clinics show no positive effect on the treatment of SFTS yet and fail to decrease the mortality rate (11), several clinical trials studies have shown that early administration of favipiravir could bring benefits in several specific subgroups patients, such as in cases with low viral load, and in cases aged 60–70 years (12, 13). In this case, the final improvement of the elderly patient may be attributed to the empirical use of favipiravir to control virus replication in the early stages of the disease.

HLH represents a life-threatening hyperinflammatory syndrome characterized by excessive phagocyte activation leading to tissue injury, progressive organ dysfunction, and fatal outcomes (14). Systemic inflammation in this disease arises from pathological overactivation and clonal expansion of three key immune cell types: NK cells, CD8 + T cells, and macrophages (14, 15). Treatments aimed at controlling the inflammatory cytokines could potentially mitigate pathological inflammatory responses (14, 15). Routinely, the judicious application of anti-inflammatory agents, including glucocorticoids and cytokine inhibitors, could serve as complementary interventions alongside primary therapies targeting the etiology of secondary HLH (14, 15). In this case, we carefully gave short-term methylprednisolone to control inflammatory response, and achieved satisfactory results. Recently, emerging cytokine-directed therapies may be more rational interventions in secondary HLH, such as anakinra (targeting IL-1β), tocilizumab (targeting IL-6), emapalumab (targeting IFN-*γ*), and ruxolitinib (targeting JAK–STAT) (16). Accordingly, prospective clinical studies are needed to demonstrate the effects of these targeted therapies in these patients with SFTS complicated with HLH.

In conclusion, early recognition of SFTS complicated with HLH is particularly important, and the preemptive application of favipiravir may improve the prognosis of these patients.

## Data Availability

The original contributions presented in the study are included in the article/supplementary material, further inquiries can be directed to the corresponding author.
